# Altered glycosylation profile of purified plasma ACT from Alzheimer’s disease

**DOI:** 10.1186/1742-4933-7-S1-S6

**Published:** 2010-12-16

**Authors:** Manuela Ianni, Marcella Manerba, Giuseppina Di Stefano, Elisa Porcellini, Martina Chiappelli, Ilaria Carbone, Federico Licastro

**Affiliations:** 1 Department of Experimental Pathology, School of Medicine, University of Bologna, 14 San Giacomo St, 40126 Bologna, Italy

## Abstract

**Background:**

Alzheimer’s disease (AD) is one of the most frequent cause of neurodegenerative disorder in the elderly. Inflammation has been implicated in brain degenerative processes and peripheral markers of brain AD related impairment would be useful. Plasma levels of alpha-1-antichymotrypsin (ACT), an acute phase protein and a secondary component of amyloid plaques, are often increased in AD patients and high blood ACT levels correlate with progressive cognitive deterioration. During inflammatory responses changes in the micro-heterogeneity of ACT sugar chains have been described.

**Methods:**

N-Glycanase digestion from Flavobacterium meningosepticum (PNGase F) was performed on both native and denatured purified ACT condition and resolved to Western blot with the purpose to revealed the ACT de-glycosylation pattern.

Further characterization of the ACT glycan profile was obtained by a glycoarray; each lectin group in the assay specifically recognizes one or two glycans/epitopes. Lectin-bound ACT produced a glyco-fingerprint and mayor differences between AD and controls samples were assessed by a specific algorithms.

**Results:**

Western blot analysis of purified ACT after PNGase F treatment and analysis of sugar composition of ACT showed significantly difference in “glyco-fingerprints” patterns from controls (CTR) and AD; ACT from AD showing significantly reduced levels of sialic acid. A difference in terminal GlcNac residues appeared to be related with progressive cognitive deterioration.

**Conclusions:**

Low content of terminal GlcNac and sialic acid in peripheral ACT in AD patients suggests that a different pattern of glycosylation might be a marker of brain inflammation. Moreover ACT glycosylation analysis could be used to predict AD clinical progression and used in clinical trials as surrogate marker of clinical efficacy.

## Background

Alzheimer’s disease (AD) is a neurodegenerative disorder clinically defined by progressive impairment of memory and cognitive functions. Brain pathology hallmarks of AD are extra-cellular amyloid plaques and intracellular neurofibrillary tangles, along with hyperactive microglia, activated astrocytes, degenerating neurons and synapsis loss [1].

Alpha-1-antichymotrypsin (ACT) is a secondary component of amyloid plaques [2]; it belongs to the superfamily of Serpins (serine protease inhibitors) and is also known as SERPINA3 [3]. ACT is synthesized in the liver and in other tissues, including lungs and brain. In the brain ACT is produced by activated astrocytes found near brain beta-amyloid (Aβ) deposits [4]. It has been suggested that ACT binds Aβ peptide and affects the rate of amyloid fibril formation *in vitro*[5,6]. Findings from mouse models of AD have also shown that ACT over-expression promotes Aβ peptide deposition in the brain of AD animal models [7] and affected their cognitive performances [8]. More recently, ACT has been shown to influence TAU protein phosphorylation and apoptosis in neuronal cells [9].

Interleukin- α (IL–1α), IL-1ß, IL-6, tumor necrosis factor α (TNF-α) and other cytokines are up-regulated and are associated with AD lesions. The inflammatory cytokines IL-1, IL-6, and TNF-α are produced by both activated microglia and astrocytes. Moreover, IL-6 and oncostatin M have been reported to modulate ACT production in brain astrocytes [10]. These data have suggested the notion that ACT might be a critical factor affecting both neurodegenerative process induced by amyliod deposition and brain inflammatory processes. The association of gene variations in ACT and other cytokine genes with the increased risk of AD has further reinforced the above hypothesis [11].

Whether peripheral levels of ACT may be of practical use, as AD biomarker or an indicator of the disease clinical progression, remains an open question. In fact, after the initial reports of increased blood and CSF ACT concentrations in AD patients [12-14], several studies measured ACT concentrations in blood samples drawn from subjects with AD, with other forms of dementias, and control subjects. Findings from these studies have produced conflicting results; some investigations confirming increased serum ACT levels [12,15,16] others showing normal ACT blood levels in AD [17,18]. Recent findings indeed showed that peripheral blood ACT levels were increased in AD patients or subjects with cognitive alteration and no dementia and high ACT levels correlated with progressive cognitive deteriorations [19]. These data paralleled other findings showing that ACT blood levels correlated with cognitive performances in elderly without dementia [20]. Different techniques for ACT detection, different criteria for the selection of controls and AD patients or small numbers of cases and controls included in the studies may account for contradictory results regarding the association of abnormal ACT plasma levels with AD. Moreover, alterations in molecular forms of ACT present in tissues and/or blood might also account for increased variability of ACT detection in AD and controls. However, no investigation has focused upon ACT molecular rearrangement in AD.

ACT plays a role in the modulation of brain amyloid deposition and immune responses, both processes are thought to be important contributors to the pathogenesis of AD [21]. Inflammatory states are usually associated with changes in the glycosylation pattern of acute phase proteins [22,23]. ACT is a glycoprotein and carbohydrates accounts approximately for 25% of its molecular weight. The sugar chain composition of ACT was studied by affinity immune-electrophoresis with Concanavalin A [24], by high resolution ^1^H-NMR spectroscopy [25] and, more recently, by mass spectrometry techniques [26]. ACT contains six N-glycosylation sites and shows four oligosaccharide side-chains with disialyl diantennary and trisialyl triantennary type glycan structures with traces of disialylated triantennary oligosaccharides. Studies from other biology fields showed that inflammatory responses causes changes in the micro-heterogeneity of ACT sugar chains. Such changes were observed in several disease states, such as prostate cancer, myocardial infarction, ovary cancer, septic inflammation, metastatic breast cancer, connective tissue disease and pulmonary sarcoidosis [24,25,27-29] .

In AD altered glycosilation pattern of presenilin-1, a molecule forming the catalytic core of the γ-secretase complex and able to generate amyloidogenic peptides [30] and an abnormal glycosylation of reelin, a glycoprotein essential for the correct cyto-architectonic organization of the developing CNS, were previously shown [31].

No data on plasma ACT glycosylation patterns in AD are on record. Here we have shown that glycosylation pattern of this molecule from the peripheral blood of AD patients and healthy controls is partially different.

## Methods

### Patients

The control plasma samples were from the “Conselice Study of brain aging” [32] and the demented patients were also from a different Northern Italy clinical longitudinal study, where AD patients were followed up for two years and their cognitive performances recorded. Patients and controls were Caucasians and informed consent from each control and AD relative was obtained.

Diagnosis of probable AD was performed according to standard clinical procedure and followed the NINCDS/ADRDA and DSM-IV-R criteria [33,34]. Cognitive performances were measured according to MMSE. Cognitive decline during the 2 year longitudinal follow up in AD patients was also assessed by the MMSE scores, according to the method suggested elsewhere [35].

### Purification of ACT from plasma of CTR and AD

Plasma samples from CTR and AD patients with comparable ACT levels were chosen. ACT levels in plasma were measured by using a competitive ELISA assay, as described elsewhere [19]. Plasma samples from 20 CTR or 19 AD patients were pooled in 2 distinct experimental sets (CTR 1 and 2 and AD 1 and 2, respectively). All experiments were performed using purified ACT obtained from these plasma sample pools.

Purification of ACT was performed by affinity chromatography using Hitrap NHS-activated HP columns (1 ml) (GE Lifesciences, Milan). 10 mg of sheep anti-human ACT antibody (AbCam, Cambridge) was coupled to the column matrix according to the manufacturer’s instructions.

Pooled plasma samples (100 μl) containing about 70 μg ACT were diluted to 10 ml with PBS, filtered through a 0.45 μm filter and applied to the column. Each sample was left re-circulating for 2h at room temperature using a peristaltic pump at a flow rate of 0.2 ml/min. Thereafter, the column was washed with 10 ml of PBS and bound ACT was eluted with 0.2 M glycine, pH 2.8; the purified protein was immediately neutralized with 5N NaOH and dialyzed against H_2_O and concentrated under reduced pressure.

### Assessment of purified ACT concentration by sandwich ELISA assay

96 well maxisorp plates (Nunc, Milan) were coated with 100 μl of sheep anti-human ACT antibody (AbCam, Cambridge), diluted 1:1000 in 50 mM Na/CO_3_ pH 8.5, incubated overnight at 4°C and washed. If not otherwise specified, washing of plates was always performed with 4 x 200 μl/well of PBS+0,05% Tween 20x (PBST) and incubation steps throughout the assay always lasted 2h, at 37°C, with shaking. After washing, plates were incubated with 100 μl/well of PBST+5% BSA and washed again.

Thereafter, 100 μl of commercially available ACT (Sigma, Milan) (dissolved in PBST + 1% BSA), in several dilutions ranging from 0 to 200 ng/ml to generate a standard curve, and test samples were added to the plate wells. After incubation and washing, plates were incubated with primary antibody (100 μl/well of rabbit anti-human ACT antibody (Dako, Milan), diluted 1:1000 in PBST+1% BSA) and secondary HRP-conjugated antibody (goat anti-rabbit-HRP (Santa Cruz, Heidelberg), diluted 1:1000 in PBST+1% BSA).

Following the usual PBST washes, an additional wash with 200 μl of PBS without Tween was performed and 100 μl of peroxidase substrate (ABTS) (Roche, Milan) diluted in ABTS buffer (Roche, Milan) was added to the wells.

Absorbance was recorded by an automatic ELISA reader at 405 nm (Biorad, Milan).

### De-glycosylation by N-Glycanase digestion of purified ACT

N-Glycanase from Flavobacterium meningosepticum (PNGase F) was used (BioLabs, Milan). De-glycosylation was performed on both native and denatured purified ACT.

Reaction in native conditions was performed by incubating 1 µg of purified ACT with 500 U of PNGase F in 50 mM sodium phosphate pH 7.5, 1% NP-40 at 37°C for 1 and 3 h.

Denaturation of purified ACT was obtained by heating the protein at 100°C for 10 min in the presence of 0.5% SDS and 40 mM dithiothreitol (DTT). After denaturation, ACT was reacted with PNGase F, as described above.

De-glycosylated ACT samples were resolved on a 10% SDS-polyacrylamide gel, blotted on a PVDF membrane,visualized by immune reaction with a specific antibody (rabbit anti-human ACT (Dako, Milan) and revealed by a Cy5-labelled secondary antibody (GE Lifesciences, Milan).

### Glycan composition analysis of purified ACT

The glycan profile of purified ACT samples was obtained by using the Qiagen Qproteome™ GlycoArray. Briefly, 5 μg of purified ACT were absorbed onto the surface of the GlycoArray slide, following the manufacturer’s instructions. Lectin-bound ACT was revealed by immune reaction using the rabbit anti-human ACT antibody (QIAGEN, Dako, Milan) and the Cy5-labelled secondary antibody (GE Lifescience, Milan). The entire process was performed in parallel without samples on a separate control array. At the end of the procedure, array slides were scanned and analyzed using the Scann Array 4000 scanner (Packard Biochip Technologies, Milan). Array image data were analyzed using the Qproteome Glycoarray Analysis Software (QIAGEN), which calculates the “glyco-fingerprint” of the sample protein by subtracting the control array signals from the experimental sample array signals. Fingerprint deconvolution was performed by algorithms using rule-based technology calibrated to a wide range of standard proteins. Each lectin group in the assay specifically recognizes one or two glycans/epitopes, although a degree of interdependence between these groups is present. This algorithm according to manufacturer calculates relative abundance of glycan epitopes and provides array-binding information on the proportion of various features within a glycoform population.

## Results

Clinical, cognitive and epidemiological variables along with number of subjects, ACT plasma levels, purified pooled ACT samples, age, gender, cognitive status assessed by MMSE scores at the time of clinical diagnosis and two years later from controls (CTR) and AD are summarized in Table 1. The AD 1 showed a higher cognitive deterioration during a 2 year follow up than the AD 2. Plasma samples from 2 different group of control (CTR 1 and 2) and AD patients (AD 1 and 2) were used for the purification of ACT and the biochemical analysis. Mean plasma ACT levels between 2 groups of controls and AD patients were comparable, as well as those of the collected ACT after the purification procedures.

**Table 1:** Epidemiological and clinical features from investigated subjects

	CTR 1	CTR 2	AD 1	AD 2
N° of samples	10	10	9	10
ACT mean (µg/ml)	763	789	695	876
ACT purify (µg/ml)	26	38	27	32
Age	73	75	80	77
Gender	9 F - 1 M	4 F – 6 M	7 F - 2 M	7 F - 3 M
Evolution	4 S- 1 I - 5 NA	2 S- 5 I- 3 NA	2 F – 6 I – 1 S	1 F – 5 I – 4 S
MMSE time 0	28	28	19	19
MMSE after 2 year follow up	27	26	13	18

Number of subjects, ACT plasma levels, purified ACT of subjects’s pool, age, gender, group of cognitively state, MMSE scores at the beginning of the study and two years later are summarized in this table.

**Figure 1:**
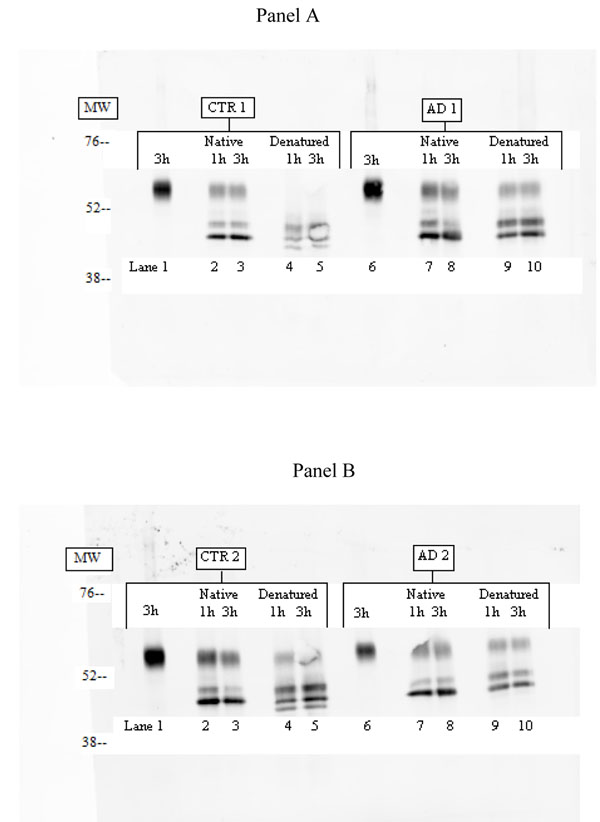
**Western blot analysis of ACT after PNGase F digestion.** Panel A. Lane 1, CTR 1 incubated 3 hours without PNGaseF; lane 2, CTR 1 in condition Native incubated for 1 h; lane 3, CTR 1 Native incubated for 3 h; lane 4, CTR 1 Denatured incubated for 1 h; lane 5, CTR 1 Denatured incubated for 3 h; lane 6, AD 1 incubated 3 hours without PNGaseF; lane 7, AD 1 Native incubated for 1 h; lane 8, AD 1 Native incubated for 3 h; lane 9, AD 1 Denatured incubated for 1 h; lane 10 AD1 Denatured incubated for 3 h. Panel B refers to CTR 2 and AD 2 with the same treatment. MW= molecular weight.

Figure 1 shows Western Blot analysis of purified ACT from CTR 1 and 2 or AD 1 and 2 treated with PNGase F. De-glycosylation of native purified ACT form both CTR and AD samples resolved into three protein bands and no qualitative differences were observed between CTR and AD. On the contrary, when PNGase F treatments was performed on denatured purified ACT, four bands were detected in both CTR 1 and 2, whereas ACT from AD samples resolved again into three bands. Semi quantitative evaluation of fluorescence band intensity from Figure 1 is reported in Table 2. Total fluorescence from ACT PAGE electrophoresis and western blot analysis in band 1 from CTR and AD was comparable (CTR 1 = 37977; AD 1 = 44469; CTR 2 = 35449; AD 2 = 23722). Results regarding total fluorescence and its percentage in band 1, 2 and 3 (native) and 1, 2, 3 and 4 (denatured) are reported in Table 2. Some difference was observed in native samples from CTR 2 and AD 2 after 3 hours of incubation with PNGase F enzyme. Under mild denaturing treatment band 4 was never detected in AD 1 and AD 2 and strong difference in fluorescence intensity in band 1, 2 and 3 after 3 hours of PNGase F digestion were found. Moreover, fluorescence intensity in band 1, 2 and 3 from AD 1 and AD 2 was quite different from those of CTR 1 and CTR 2, especially after 3 hour treatment. In fact, in these condition both total fluorescence intensity and its percentage, were higher in bands 1 and 2 from AD 1 and AD 2 samples than those detected in CTR 1 and/or CTR 2.

**Table 2:** Fluorescence intensity analysis after PNGase F treatment

PANEL A	CTR 1				AD 1			
Band	Native 1 1 h	Native 3 3 h	Denatured 1 h	Denatured 3 h	Native 1 1 h	Native 3 3 h	Denatured 1 h	Denatured 3 h
1	12271 (41.2%)	11658 (38.6%)	8608 (57%)	978 (5.5%)	20528 (40.5%)	16066 (37.4%)	10821 (30.5%)	10480 (25.3%)
2	5297 (17.8%)	5272 (17.4%)	4313 (28%)	9264 (52.6%)	10135 (20%)	5651 (13.1%)	12959 (36.6%)	15418 (37.1%)
3	12229 (41%)	13257 (44%)	2240 (15%)	4689 (26.6%)	19972 (39.5%)	21315 (49.5%)	11668 (32.9%)	15594 (37.6%)
4				2702 (15.3%)				

PANEL B	CTR 2				AD 2			

Band	Native 1 1 h	Native 3 3 h	Denatured 1 h	Denatured 3 h	Native 1 1 h	Native 3 3 h	Denatured 1 h	Denatured 3 h
1	18537 (46.7%)	12721 (44.9%)	6925 (25.1%)	5234 (15.7%)	10371 (44.4%)	10937 (37.2%)	6649 (37.1%)	5579 (36.7%)
2	6293 (15.9%)	2825 (10%)	11136 (40.4%)	14381 (43.3%)	2916 (12.5%)	4048 (13.8%)	5527 (30.8%)	3913 (25.8%)
3	14817 (37.4%)	12804 (45.1%)	6094 (22.1%)	10005 (30.1%)	10083 (43.1%)	14385 (49%)	5759 (32.1%)	5706 (37.5%)
4			3428 (12.4%)	3632 (10.9%)				

Band fluorescence intensity after PNGase F treatment (see Western Blot bands in figure 1, panels A and B).

Further analysis of sugar composition in purified ACT from CTR 1 and 2 or AD 1 and 2 was performed by using the Qproteome™ GlycoArray kit. This glyco-array consisted of 24 lectins covering a large pattern of glycan specificity. Binding of a glycoprotein to the array results in a characteristic fingerprint pattern that is highly sensitive to the glycan structure and composition. Glycan structure semi-quantitatively detectable by the array include: N-glycans (bi-antennary, tri/tetra antennary, high mannose, sialic acid, terminal N-acetyl glucosamine (GlcNac), terminal N-acetyl galactosamine (GalNac) and bisecting GlcNac and presence or absence of O-glycans. The fingerprint is interpreted by proprietary knowledge-based algorithms to produce the glycoanalysis results, a list of epitopes and their relative abudance.

Fingerprint data, analyzed by the Qproteome™ GlycoArray software, produced a detailed profile of ACT glycosylation status and a glycan epitope prediction pattern by the specific algorithm (Table 3). The Qproteome™ GlycoArray method provides four levels quantification output for most epitopes: not Detected=up to 10%, low=11-30%, medium=31-70%, high=71-100% and a qualitative glycan profile for other epitopes (detected/not detected). Quantitative difference in purified ACT lectin reactivity between the experimental sets, i.e. Purified ACT from AD 1 and 2 showed significantly reduced levels of sialic acid when compared to those from CTR 1 and 2. Moreover, a difference in terminal GlcNac residues was found between AD 1 and AD 2 groups. It is interesting to note that AD 1 showed a faster cognitive deterioration than AD 2 in a 2 years follow up. In fact, as shown in Table 1, AD 1 patients loosed 5 points in the MMSE score and AD 2 patients only 1 point.

**Table 3:** Glycan epitope pattern of Cy5 labeled ACT

Glycan epitope	CTR 1	AD 1	CTR 2	AD 2
N-linked:				
Bi Antennary	Not Detected	Not Detected	Not Detected	Not Detected
Tri/Tetra Antennary	High	High	High	High
High Mannose	Not Detected	Not Detected	Not Detected	Not Detected
Sialic Acid	High	Medium	High	Medium
Terminal GlcNAc	Low	Low	Low	Not Detected
Terminal GalNAc	Not Detected	Not Detected	Not Detected	Not Detected
Bisecting GlcNAc	Not Detected	Not Detected	Not Detected	Not Detected
O-Glycans	Not Detected	Not Detected	Not Detected	Not Detected

Glycan profile produced for ACT using the Qproteome Glycoarray method.

ND=not detected (up to 10%); Low=11-30%; Medium=31-70%; High=71-100%

## Discussion

Glycosylation is a versatile biochemical mechanism and one of the most abundant post-translational modification of protein; however, glycosylation of proteins is not a template driven process, is difficult to predict [36] and affects molecule stability, resistance to proteolysis, solubility and molecule functional activity. Therefore, this protein modification may play a role in affecting biological activity of molecules with a special role in the metabolic events related to neuro-degeneration and AD.

ACT is a glycoprotein and carbohydrate content reach 24% of molecular weight. This acute phase proteins is mainly synthesized by the liver, however, other tissues are able to produce and release this molecule. In fact, astrocytes synthesized and release ACT and increased levels of this protein have been found in the brain, CSF and blood from AD patients [15,19,37]. ACT levels in the blood markedly increases after tissue damages or infections [38]. We already postulated that a proportion of plasma ACT in AD might derive from the brain as a by-product of neurodegenerative processes and inflammation in the central nervous system [39]. As for other glycoproteins, micro-heterogeneity of ACT may be ascribed to differences in carbohydrate structure and indeed different patterns of ACT micro-heterogeneity has been shown in different diseases [40,41].

To obtain usable level of purified ACT, samples from AD or control were pooled; plasma samples showing comparable levels of this serpin, as assessed by competitive ELISA, i.e. moderately high ACT levels, were chosen. This step is relevant, since plasma levels of ACT and other serpins increase in different pathological conditions; however, in this investigation both patients controls were free from cancer, infections and inflammatory diseases.

Here we showed that after partial denaturation, purified ACT from AD plasma samples were less sensitive to enzymatic digestion by N-glycanase than ACT from plasma samples of healthy donors. This first observation suggested a different glycosylation pattern in ACT form AD patients, since denaturation was shown to increase deglycosylation by glycanase [25]. Different deglycosylation patterns of denatured ACT between AD and CTR may be ascribed to differentially presence of fucose residues linked α(1-3) to ASN bound N-acetylglucosamine that resistant to PNGase F action.

Purified ACT was then analysed by a lectin array specifically developed for investigating protein glycan content and composition [42]. This analysis resulted in a partially different pattern of glycan profiles between ACT from AD and controls; sialic acid content being different between AD and CTR.

This alteration may have several explanations. For instance, a proportion of circulating ACT in AD plasma may derive from other tissues than liver, possibly the brain and this molecules might show a different glycosylation signature.

On the other hand, we can not exclude another interpretation suggesting that altered ACT glycan profile from AD samples may reflect a generalized impairment of glycosylation processes involving other glycoproteins. In fact, it has previously shown that reelin, a glycoprotein essential for the correct cyto-architecture organisation of developing brain and involved in signalling pathways linked to neuro-degeneration in several human diseases, were increased in the brain from neurological disorders and showed a different glycosylation patterns in plasma from AD [31]. Moreover, acetylcholine esterase from AD samples analyzed by lectin binding activity showed different binding properties when compare with those from controls [43].

Also our data showed a slight but significant difference in the two AD sets. AD 1 showing higher fluorescence intensity in terminal GlcNac and sialic acid than AD 2. Patients belonging to the AD 1 group showed a faster cognitive deterioration rate in a 2 year follow up. Overall our data suggest altered sialic acid content in ACT from AD samples and the potential presence of focuse residues in the denatured ACT from CTR than AD samples.

## Conclusion

Altered glycosylation pattern in purified ACT from the peripheral blood of AD maight be ascribed to an increased inflammation of the brain or an altered glycation process of ACT along with several other brain proteins in AD.

Our findings suggest that low content of terminal GlcNac glycans and sialic acid in peripheral ACT might be a marker of diseases progression and it might be used in clinical trials as surrogate marker of clinical efficacy.

## Competing interests

The authors declare that they have no competing interests.

## Authors’ contribution

MI and MM performed ACT ELISA assay and ACT GlycoArray;

EP,MC and IC purification of ACT and writing article;

GDS and FL Conception and Design and writing article

## List of abbreviations

AD: alzheimer’s disease; ACT: alpha-1-antichymotrypsin; CTR: controls; IL1α: Interleukin 1α; IL-1ß: Interleukin 1ß; IL-6: Interleukin 6; TNF-α: tumor necrosis factor α; Aβ: beta-amyloid; CSF: cerebrospinal fluid; PNGase F: N-Glycanase digestion from Flavobacterium meningosepticum; GlcNac: N-acetyl glucosamine; GalNac: terminal N-acetyl galactosamine; Gal/GalNac: Galactose/N-acetyl galactosamine.
